# A high‐resolution cross‐species comparative analysis of the subchondral bone provides insight into critical topographical patterns of the osteochondral unit

**DOI:** 10.1002/ctm2.745

**Published:** 2022-02-27

**Authors:** Jana Christin Michaelis, Tamás Oláh, Steffen Schrenker, Magali Cucchiarini, Henning Madry

**Affiliations:** ^1^ Center of Experimental Orthopaedics Saarland University Homburg Germany; ^2^ Cartilage Net of the Greater Region Homburg Germany

Dear Editor,

Deciphering the hierarchical and species‐specific topographical structure of the subchondral bone has far‐reaching implications. The subchondral bone is in the focus of many pathological conditions, including osteoarthritis (OA). Location‐dependent OA development,^1^ topographical differences within individual subregions,^2^, ^3^ all influenced by the meniscus coverage^2^, ^3^ highlight the urgent need to precisely reproduce pathological alterations at high quantitative detail in appropriate in vivo models.^4^, ^5^ We performed a detailed comprehensive analysis of the zonal characteristics of the subchondral bone of mice, rats, rabbits, minipigs, and sheep, the most common animal models in orthopaedic research, applying the human tibial plateau as a model (Figure 1A), to identify the species with the highest morphological agreement.

**FIGURE 1:**
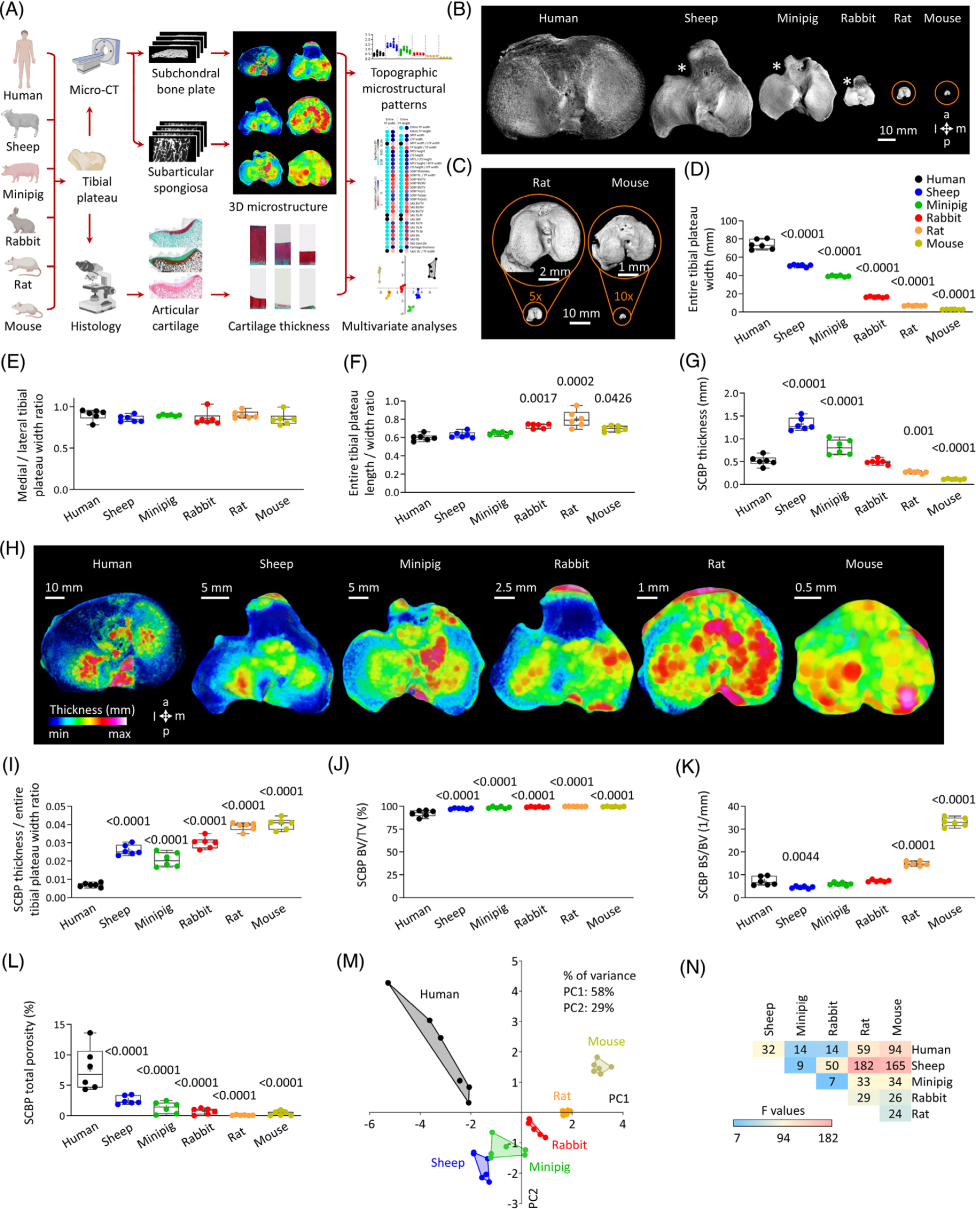
Macroscopic dimensions of the tibial plateau and comparison of the subchondral bone plate (SCBP) microstructure of six species. (A) Schematic figure (images created with BioRender.com) of the study design. (B,C) Representative 3D reconstructed micro‐CT models of human, sheep, minipig, rabbit, rat, and mouse tibial plateaus (asterisks: groove of the extensor digitorum longus tendon). Box plots (boxes: 75th–25th percentiles, whiskers: minimum and maximum, middle line: median, +: mean, dots: individual data points) of the (D) entire tibial plateau width, (E) medial/lateral tibial plateau width ratio, (F) entire tibial plateau length/width ratio, (G) SCBP thickness. (H) Representative colour‐coded 3D reconstructed micro‐CT models of SCBP thickness of the tibial plateaus of six species. Box plots of the SCBP (I) thickness normalized to the entire tibial plateau width, (J) percent bone volume (BV/TV), (K) bone surface‐to‐volume ratio (BS/BV), and (L) total porosity. (M) Principal components analysis and (N) PERMANOVA (higher F values indicate the larger difference between the groups; all *p* ≤ .042, except between rabbit and minipig *p* = .1575 and rat and mouse *p* = .0525) of the SCBP parameters. Data points represent individual samples. Abbreviations: a, anterior; d, distal; l, lateral; m, medial; p, posterior, pr, proximal. *n* = 6 per species. *p‐*Values above the box plots show comparisons to human and were determined with ANOVA or Kruskal–Wallis ANOVA

Macroscopic morphology was largely conserved (Figure 1B–F; Figure S1). The groove of the extensor digitorum longus tendon was present only in sheep, minipigs, and rabbits (Figure 1B,C; Figure S1). Normalized ratios of anatomical landmarks were highly similar in the larger species (Figure 1D–F; Figure S1). Humans had an extremely thin, porous and less compact subchondral bone plate (SCBP), similar in many characteristics to rabbits and minipigs, strikingly different from rats and mice (Figure 1G–N; Figure S2). Solely in larger species (humans, sheep, minipigs; partly in rabbits), the SCBP was thinner under the meniscus‐covered peripheral areas than in the not meniscus‐covered central area (Figure 1H), a finding highly relevant for translational studies, for example, examining OA induction by meniscus tears. Of note, mice and rats did not exhibit such pattern. Multivariate analyses of all SCBP parameters characterized minipigs and rabbits as most similar to humans, followed by sheep, rats and mice (Figure 1M,N; Figure S2D).

Human OA induces distinct degrees of architectural alterations in the subarticular spongiosa.^2^, ^3^ Species with larger joint size, especially humans, had a lower relative bone surface with fewer, thicker subarticular trabeculae in a less connected and complex arrangement (Figure 2A–L). Gait‐specific^4^, ^5^ biomechanical forces and human sedentary lifestyle may explain such structural adaptations. Rats and mice significantly differed in most parameters from humans (Figure 2A–L). Multivariate analyses indicated that the structurally weaker human subarticular spongiosa is matched by no other species, although minipigs, rabbits, and sheep offer compromises (Figure 2M–O).

**FIGURE 2:**
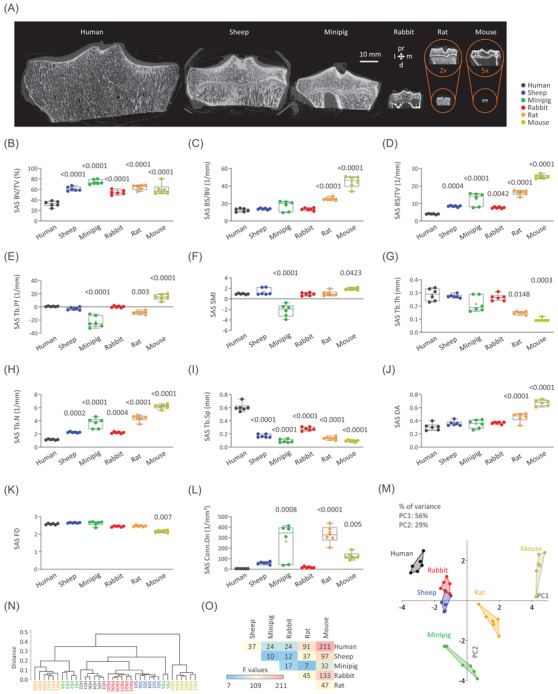
Comparison of the subarticular spongiosa microstructure of six species. (A) Representative 2D micro‐CT images of human, sheep, minipig, rabbit, rat, and mouse tibial plateaus. Box plots of the (B) percent bone volume (BV/TV), (C) bone surface‐to‐volume ratio (BS/BV), (D) bone surface density (BS/TV), (E) trabecular pattern factor (Tb.Pf), (F) structure model index (SMI), (G) trabecular thickness (Tb.Th), (H) trabecular number (Tb.N), (I) trabecular separation (Tb.Sp), (J) degree of anisotropy (DA), (K) fractal dimension (FD), and (L) connectivity density (Conn.Dn) of the subarticular spongiosa (SAS). (M) Principal components analysis, (N) cluster analysis, and (O) PERMANOVA (all *p* ≤ .0465) of the subarticular spongiosa parameters. Data points represent individual samples. Abbreviations: d, distal; l, lateral; m, medial; pr, proximal. *n* = 6 per species. *p‐*Values above the box plots show comparisons to human and were determined with ANOVA or Kruskal–Wallis ANOVA

Meniscal coverage dictates a topographical pattern of the SCBP microstructure that becomes disturbed in advanced OA.^2^ Detailed analysis of the tibial plateaus identified a denser and more solid bone structure in the more exposed central locations (not covered by menisci) in humans, sheep, minipigs mostly laterally (Figure 3B–E; Figure S3). It is possible that larger species depend more on the osteochondroprotective effect of the menisci on the (lateral) SCBP. In contrast, rabbits, rats, and mice lack such adaptation (Figure 3B–E; Figure S3). The human subarticular spongiosa is sclerotic in not meniscus‐covered subregions.^6^ We identified an expanded trabecular structure only in minipigs at these locations (Figure 3F–N; Figure S4). In contrast, the trabecular network of sheep, rabbits, and partly also humans is reduced at the central region, with an uncertain relation to meniscal coverage (Figure 3F–N; Figure S4). If characteristic topographical patterns according to meniscal coverage are of importance, only sheep and minipigs reflect the human situation. The marked differences of small rodents discourage their use as realistic models to reflect the human subchondral trabecular structure and meniscal structural patterns.

**FIGURE 3:**
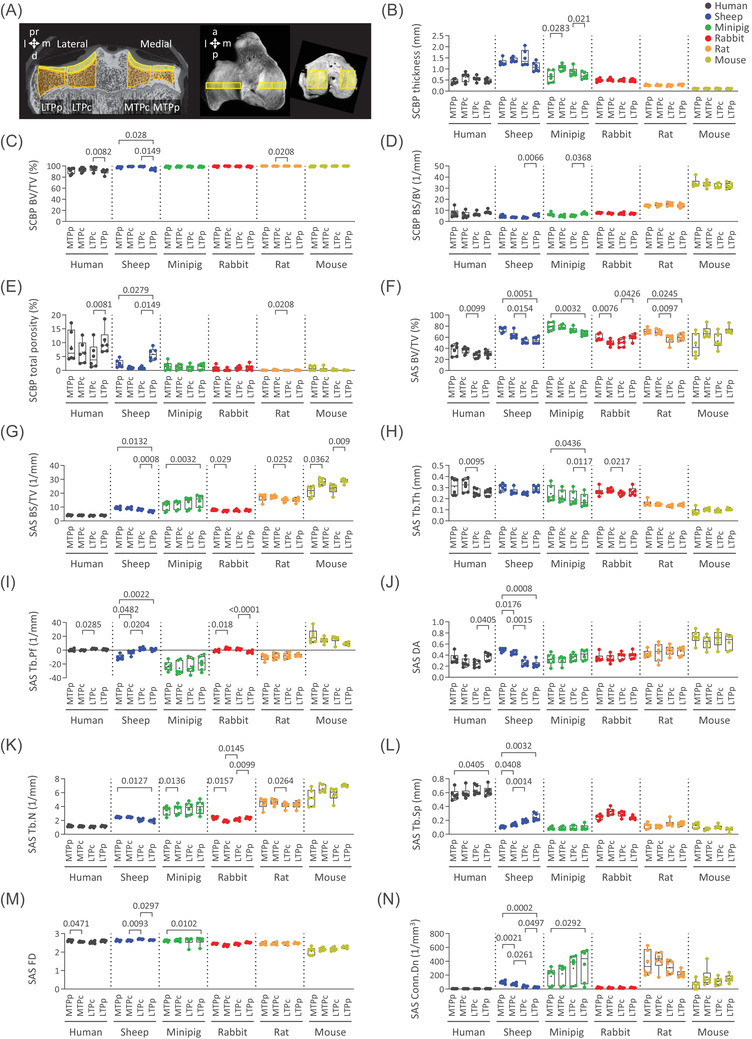
Detailed regional analysis of the subchondral bone plate and subarticular spongiosa microstructure in six species. (A) Representative 2D micro‐CT image of a sheep, and 3D reconstructed micro‐CT models of a sheep and a mouse tibial plateau showing the volumes of interests used for the regional analyses. Box plots of the (B) thickness, (C) percent bone volume (BV/TV), (D) bone surface‐to‐volume ratio (BS/BV), and (E) total porosity of the subchondral bone plate (SCBP), and the (F) BV/TV, (G) bone surface density (BS/TV), (H) trabecular thickness (Tb.Th), (I) trabecular pattern factor (Tb.Pf), (J) degree of anisotropy (DA), (K) trabecular number (Tb.N), (L) trabecular separation (Tb.Sp), (M) fractal dimension (FD), and (N) connectivity density (Conn.Dn) of the subarticular spongiosa (SAS) in four regions. Abbreviations: a, anterior; d, distal; l, lateral; m, medial; p, posterior; pr, proximal; LTPc, lateral tibial plateau central; LTPp, lateral tibial plateau peripheral; MTPc, medial tibial plateau central; MTPp, medial tibial plateau peripheral. *n* = 6 per species. *p‐*Values above the box plots were determined with RM‐ANOVA or Friedman test. Only the relevant comparisons (i.e. MTPp vs. MTPc, LTPp vs. LTPc, MTPp vs. LTPp, MTPc vs. LTPc) were performed

Lateral‐to‐medial differences exist in humans, sheep, and rats. The more complex and solid subarticular spongiosa of the medial tibial plateau is largely absent in rabbits and mice (Figure 3F–N; Figure S4). Minipigs, in contrast, had expanded trabeculae in the lateral tibial plateau. Since the medial tibiofemoral compartment is involved in 67% of all OA cases, and the load distribution can be (non)surgically modified,^7^ such lateral‐medial dissimilarities are of major translational relevance. Sheep or rats reflect these patterns, the former are better suited for precision surgical interventions.^7^

Chondrocytes were always typically organized, except in the extremely thin mouse cartilage (Figure 4A,B). Absolute cartilage thickness decreased towards species with smaller body size in a simple^8^ or a negative allometric relationship.^9^, ^10^ When normalized to tibial plateau width, only rabbits displayed an outstandingly large relative cartilage thickness. Of special translational importance, we detected thicker cartilage in the not meniscus‐protected central subregions in humans, sheep, minipigs, and rabbits, but not in small rodents (Figure 4A–F; Figure S5A). The similarity to humans was ranked in decreasing order: Sheep, minipigs, rabbits, rats, mice considering all evaluated parameters (Figure 4G,H; Figure S5B), supporting their applicability as models to study subchondral bone alterations.

**FIGURE 4:**
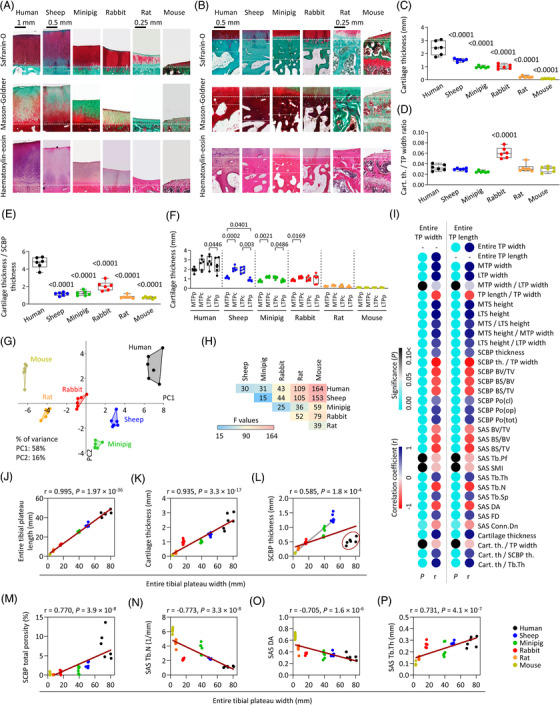
Histology and correlations of the osteochondral unit and multivariate analysis of all examined parameters. Safranin O/fast green, Masson–Goldner trichrome and haematoxylin‐eosin stained histological sections of human, sheep, minipig, rabbit, rat, and mouse tibial plateaus showing the (A) articular cartilage and the (B) subchondral bone. Dashed lines indicate the alignment of the images according to the cement line, dotted lines indicate the border between the subchondral bone plate (SCBP) and subarticular spongiosa. (C) Articular cartilage thickness was measured with micro‐CT. Articular cartilage thickness normalized to the (D) entire tibial plateau (TP) width, and (E) SCBP thickness. *p‐*Values above the box plots show comparisons to human and were determined with ANOVA. (F) Detailed regional analysis of the subarticular spongiosa microstructure in six species. *p‐*Values above the box plots were determined with RM‐ANOVA or Friedman test. (G) Principal components analysis and (H) PERMANOVA (all *p* ≤ .048) of all data, including cartilage thickness, tibial plateau dimensions and subchondral bone parameters, were evaluated in the study. Data points represent individual samples. (I) Pearson correlation matrix of the *p*‐values and the correlation coefficients (*r*) of the examined osteochondral parameters compared with the entire tibial plateau (TP) width and length. Scatter plot and linear regression of the Pearson correlation between entire tibial plateau width and (J) entire tibial plateau length, (K) articular cartilage thickness, (L) SCBP thickness (dark red ellipse: outlying human data; grey line: linear regression of only the quadruped animals, excluding the human data; *r* = 0.961, *p* = 1.8 × 10^–17^), (M) SCBP total porosity, (N) subarticular spongiosa (SAS) trabecular number (Tb.N), (O) SAS degree of anisotropy (DA), and (P) SAS trabecular thickness (Tb.Th). *n* = 6 per species. Abbreviations: LTP, lateral tibial plateau; LTS, lateral tibial spine; MTP, medial tibial plateau; MTS, medial tibial spine; th., thickness

Considering all species together, most osteochondral parameters presented strong and significant correlations with joint size (Figure 4I–P; Figure S6),^9^, ^10^ suggesting that the relation between these metrics is conserved across species. Interestingly, due to the extreme thinness of the human SCBP, the correlation between the SCBP thickness and joint size was only moderate when all species (including humans) were considered. Excluding humans, correlation became very strong (Figure 4L).

Limitations are that morphological parameters of the osteochondral unit of only one strain of each species were included. Functional, cellular, and physiological characteristics were not examined. Due to the extreme differences in joint sizes, scanning resolution standardization was impossible.

In summary, our study makes five major contributions to better capture the topographical subchondral bone cross‐species complexity: (i) The human SCBP is remarkably thin and porous compared to larger animals, similar in many characteristics to minipigs and rabbits, distinctly different from small rodents. (ii)The subarticular trabecular structure of all animal species is more dense and complex than humans. (iii) Regional patterns in the SCBP according to meniscal coverage exist only in larger species (humans, sheep, minipigs). The subarticular spongiosa displays structural lateral‐to‐medial differences in humans, sheep, rats and minipigs, largely absent in rabbits and mice. (iv) Most of the osteochondral parameters show strong and significant correlations with joint size. (v) The declining rate of analogy in macroscopic anatomy and microstructure of the tibial plateau of the animal species to humans is: sheep ≈ minipigs > rabbits > > rats > > mice. Together this comparative investigation closes major gaps in our understanding of the cross‐species topographical patterning of subchondral bone that will be critical for future clinical and translational approaches.

## CONFLICT OF INTEREST

HM received grants from DFG (German Research Foundation), Deutsche Arthrose‐Hilfe, Deutsche Gesellschaft für Orthopädie und Orthopädische Chirurgie, Universität Sorbonne Paris, and Fidia farmaceutici S.p.A., speaker fees, and travel fees from Novartis and Fidia farmaceutici S.p.A., is a member of the Scientific Advisory Board of Thuasne and Bone therapeutics and received travel fees and was a member of the Scientific Advisory Board of Kolon TissueGene. All other authors declare no conflict of interest.

## Supporting information

Supporting Information

Supporting Information
