# Supplementary material for: A high‐resolution cross‐species comparative analysis of the subchondral bone provides insight into critical topographical patterns of the osteochondral unit
Source: Clin Transl Med. 2022 Feb 27;12(2):e745. doi: 10.1002/ctm2.745 (PMC8882244; doi:10.1002/ctm2.745)
Supplement: Supplementary file 1 — Supporting Information [file CTM2-12-e745-s002.docx]

SUPPLEMENTARY INFORMATION

for

**A high-resolution cross-species comparative analysis of the subchondral bone provides insight into critical topographical patterns of the osteochondral unit**

Jana Christin Michaelis^1,#^, Tamás Oláh^1,2,#^, Steffen Schrenker^1^, Magali Cucchiarini^1,2^, Henning Madry^1,2,*^

^1^Center of Experimental Orthopaedics, Saarland University, Homburg, Germany, 66421

^2^Cartilage Net of the Greater Region, Homburg, Germany, 66421

#: These authors contributed equally: Jana Christin Michaelis and Tamás Oláh.

** Corresponding author:*

Henning Madry, Center of Experimental Orthopaedics, Saarland University; Kirrberger Straße 100, Building 37, D-66421 Homburg/Saar, Germany. Phone: +49-6841-1624590. Fax: +49-6841-1624988. E-mail: [henning.madry@uks.eu](mailto:henning.madry@uks.eu)

**List of Supplementary Materials:**

Supplementary Table 1

Supplementary Figures S1-S6

Supplementary Data 1

Supplementary Background

Supplementary Methods

Supplementary Discussion

Supplementary References

**SUPPLEMENTARY TABLE**

**Supplementary Table 1.** Mean ± SD of the examined parameters. *n* = 6 per species.

|  | **Human** | **Sheep** | **Minipig** | **Rabbit** | **Rat** | **Mouse** |
| --- | --- | --- | --- | --- | --- | --- |
| **Cartilage and tibial plateau morphology** |  |  |  |  |  |  |
| Entire tibial plateau length (mm) | 44.42 ± 2.88 | 32.02 ± 1.81 | 25.46 ± 0.75 | 11.99 ± 0.73 | 5.60 ± 0.85 | 1.97 ± 0.15 |
| Entire tibial plateau width (mm) | 73.98 ± 5.43 | 50.85 ± 1.23 | 39.35 ± 0.74 | 16.44 ± 0.63 | 6.94 ± 0.32 | 2.82 ± 0.13 |
| Medial tibial plateau width (mm) | 30.54 ± 4.18 | 19.69 ± 0.72 | 15.79 ± 0.56 | 6.17 ± 0.45 | 2.52 ± 0.13 | 0.94 ± 0.06 |
| Lateral tibial plateau width (mm) | 33.79 ± 3.63 | 23.05 ± 0.81 | 17.66 ± 0.71 | 7.23 ± 0.52 | 2.81 ± 0.21 | 1.11 ± 0.09 |
| Medial / lateral tibial plateau width ratio | 0.90 ± 0.06 | 0.86 ± 0.04 | 0.89 ± 0.01 | 0.86 ± 0.09 | 0.90 ± 0.04 | 0.85 ± 0.08 |
| Entire tibial plateau length / width ratio | 0.60 ± 0.04 | 0.63 ± 0.03 | 0.65 ± 0.02 | 0.73 ± 0.03 | 0.80 ± 0.09 | 0.70 ± 0.03 |
| Medial tibial spine height (mm) | 10.45 ± 1.45 | 6.27 ± 0.38 | 4.42 ± 1.01 | 1.23 ± 0.19 | 0.34 ± 0.05 | 0.13 ± 0.02 |
| Lateral tibial spine height (mm) | 9.45 ± 1.54 | 6.71 ± 0.94 | 5.64 ± 0.71 | 1.64 ± 0.16 | 0.56 ± 0.11 | 0.18 ± 0.01 |
| Medial / lateral tibial spine height ratio | 1.12 ± 0.17 | 0.94 ± 0.09 | 0.78 ± 0.11 | 0.75 ± 0.08 | 0.61 ± 0.13 | 0.71 ± 0.08 |
| Medial tibial spine height / medial tibial plateau width ratio | 0.34 ± 0.02 | 0.32 ± 0.02 | 0.28 ± 0.06 | 0.20 ± 0.04 | 0.13 ± 0.02 | 0.14 ± 0.03 |
| Lateral tibial spine height / lateral tibial plateau width ratio | 0.28 ± 0.04 | 0.29 ± 0.03 | 0.32 ± 0.03 | 0.23 ± 0.03 | 0.20 ± 0.02 | 0.17 ± 0.02 |
| Cartilage thickness / Entire tibial plateau width ratio | 0.03 ± 0.01 | 0.03 ± 0.00 | 0.02 ± 0.00 | 0.06 ± 0.01 | 0.03 ± 0.01 | 0.03 ± 0.01 |
| SCBP thickness / Entire tibial plateau width ratio | 0.01 ± 0.00 | 0.03 ± 0.00 | 0.02 ± 0.00 | 0.03 ± 0.00 | 0.04 ± 0.00 | 0.04 ± 0.00 |
| Cartilage thickness / SCBP thickness ratio | 4.73 ± 0.65 | 1.15 ± 0.19 | 1.23 ± 0.28 | 2.06 ± 0.52 | 0.82 ± 0.19 | 0.73 ± 0.10 |
| Cartilage thickness / SAS trabecular thickness ratio | 8.55 ± 1.36 | 5.47 ± 0.52 | 4.83 ± 1.39 | 3.85 ± 0.82 | 1.51 ± 0.35 | 0.89 ± 0.22 |
| Cartilage thickness (mm) | 2.43 ± 0.53 | 1.50 ± 0.11 | 0.98 ± 0.07 | 1.00 ± 0.17 | 0.22 ± 0.06 | 0.08 ± 0.02 |
| **Subchondral bone plate** |  |  |  |  |  |  |
| SCBP thickness (mm) | 0.52 ± 0.11 | 1.32 ± 0.14 | 0.82 ± 0.17 | 0.49 ± 0.05 | 0.27 ± 0.02 | 0.11 ± 0.01 |
| SCBP BV/TV (%) | 92.35 ± 3.58 | 97.46 ± 0.68 | 98.74 ± 1.03 | 99.33 ± 0.55 | 99.92 ± 0.07 | 99.65 ± 0.39 |
| SCBP BS/BV (1/mm) | 7.20 ± 1.94 | 4.51 ± 0.53 | 6.03 ± 0.68 | 7.30 ± 0.44 | 14.83 ± 1.05 | 33.08 ± 1.98 |
| SCBP BS/TV (1/mm) | 6.54 ± 1.49 | 4.37 ± 0.48 | 5.95 ± 0.66 | 7.25 ± 0.45 | 14.77 ± 1.08 | 32.97 ± 1.98 |
| SCBP Po(cl) (%) | 0.11 ± 0.07 | 0.49 ± 0.12 | 0.31 ± 0.26 | 0.00 ± 0.01 | 0.00 ± 0.00 | 0.00 ± 0.00 |
| SCBP Po(op) (%) | 7.54 ± 3.60 | 2.06 ± 0.68 | 0.95 ± 0.81 | 0.67 ± 0.54 | 0.08 ± 0.07 | 0.35 ± 0.39 |
| SCBP Po(tot) (%) | 7.65 ± 3.58 | 2.55 ± 0.68 | 1.30 ± 0.97 | 0.72 ± 0.49 | 0.08 ± 0.07 | 0.35 ± 0.39 |
| **Subarticular spongiosa** |  |  |  |  |  |  |
| SAS BV/TV (%) | 32.72 ± 5.39 | 61.12 ± 4.09 | 74.36 ± 4.39 | 54.66 ± 4.43 | 64.24 ± 4.91 | 59.87 ± 10.53 |
| SAS BS/BV (1/mm) | 12.55 ± 1.95 | 13.88 ± 0.84 | 17.33 ± 5.86 | 13.53 ± 1.55 | 25.34 ± 1.68 | 44.89 ± 6.55 |
| SAS BS/TV (1/mm) | 4.00 ± 0.25 | 8.49 ± 0.43 | 12.52 ± 3.72 | 7.63 ± 0.48 | 16.15 ± 1.42 | 25.34 ± 1.14 |
| SAS Tb.Pf (1/mm) | 0.71 ± 0.58 | -2.71 ± 2.12 | -22.81 ± 8.79 | 0.18 ± 1.36 | -8.63 ± 2.49 | 14.9 ± 4.37 |
| SAS SMI | 0.95 ± 0.11 | 1.36 ± 0.67 | -2.18 ± 1.16 | 0.92 ± 0.30 | 1.02 ± 0.49 | 1.90 ± 0.21 |
| SAS Tb.Th (mm) | 0.28 ± 0.04 | 0.28 ± 0.01 | 0.22 ± 0.06 | 0.26 ± 0.03 | 0.15 ± 0.01 | 0.09 ± 0.01 |
| SAS Tb.N (1/mm) | 1.14 ± 0.08 | 2.25 ± 0.08 | 3.66 ± 0.76 | 2.19 ± 0.12 | 4.38 ± 0.48 | 6.17 ± 0.33 |
| SAS Tb.Sp (1/mm) | 0.61 ± 0.07 | 0.17 ± 0.02 | 0.09 ± 0.03 | 0.27 ± 0.03 | 0.14 ± 0.02 | 0.09 ± 0.01 |
| SAS DA | 0.31 ± 0.05 | 0.36 ± 0.04 | 0.35 ± 0.06 | 0.37 ± 0.02 | 0.46 ± 0.07 | 0.67 ± 0.05 |
| SAS FD | 2.58 ± 0.04 | 2.65 ± 0.02 | 2.62 ± 0.13 | 2.44 ± 0.03 | 2.46 ± 0.04 | 2.16 ± 0.06 |
| SAS Conn.Dn (1/mm^3^) | 5.08 ± 0.82 | 57.98 ± 12.59 | 262.55 ± 175.41 | 17.3 ± 6.54 | 329.99 ± 80.00 | 124.62 ± 34.13 |

**SUPPLEMENTARY FIGURES**

**
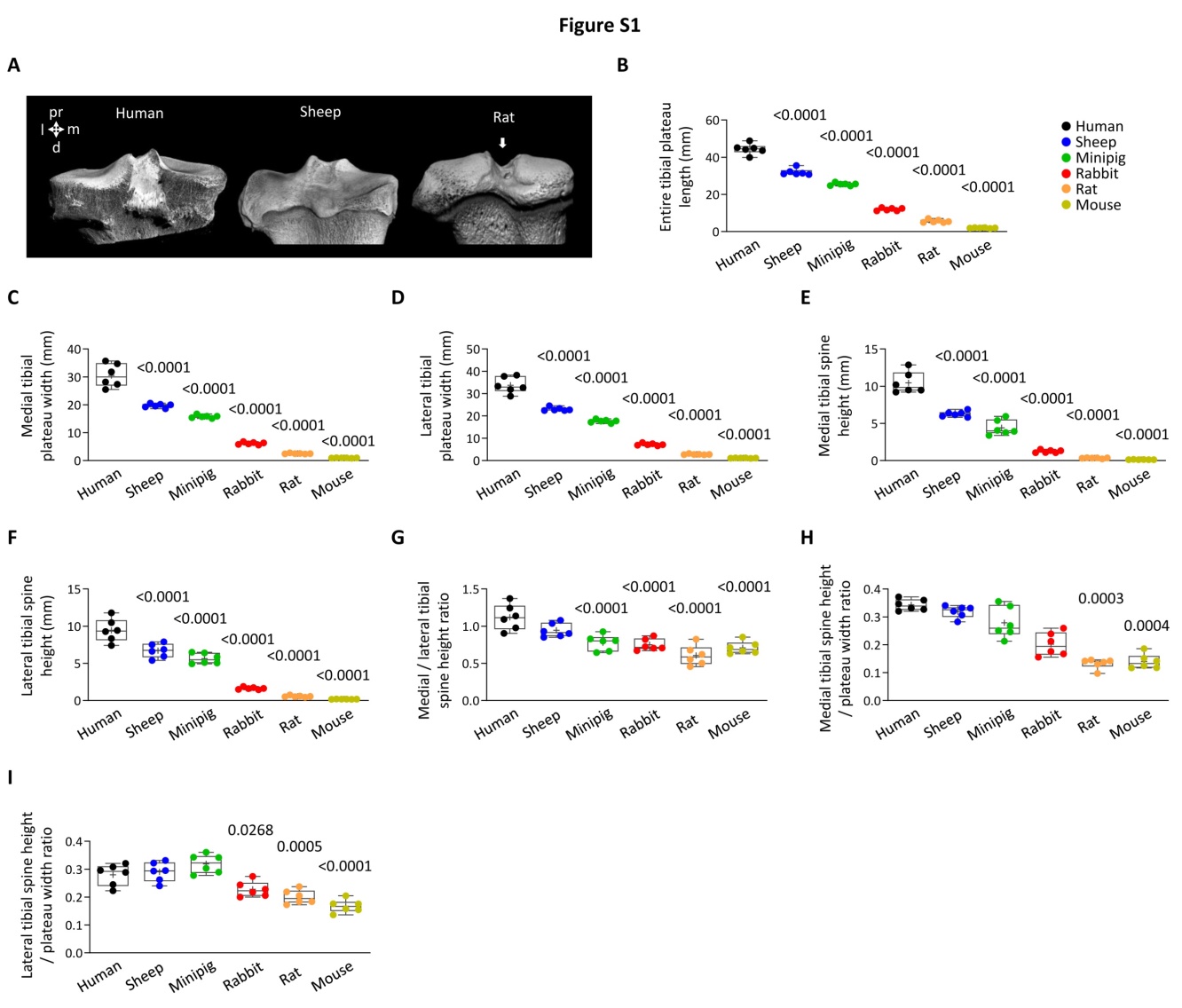
**

**Figure S1.** Macroscopic characterization of the tibial plateau in 6 mammalian species. **(A)** Representative 3D reconstructed micro-CT models (posterior view) of human, sheep and rat tibial plateaus (arrow: intercondylar fossa). Box plots (boxes: 75^th^ - 25^th^ percentiles, whiskers: minimum and maximum, middle line: median, +: mean, dots: individual data points) of the **(B)** entire tibial plateau length, and **(C)** medial, and **(D)** lateral tibial plateau width, **(E)** medial and **(F)** lateral tibial spine height, **(G)** medal / lateral tibial spine height ratio, and ratios of the **(H)** medial and **(I)** lateral tibial spine heights to the corresponding tibial plateau widths. Data points represent individual samples. Abbreviations: d, distal; l, lateral; m, medial; pr, proximal. *n* = 6 per species. *P* values above the box plots show comparisons to human with ANOVA or Kruskal-Wallis ANOVA.

**
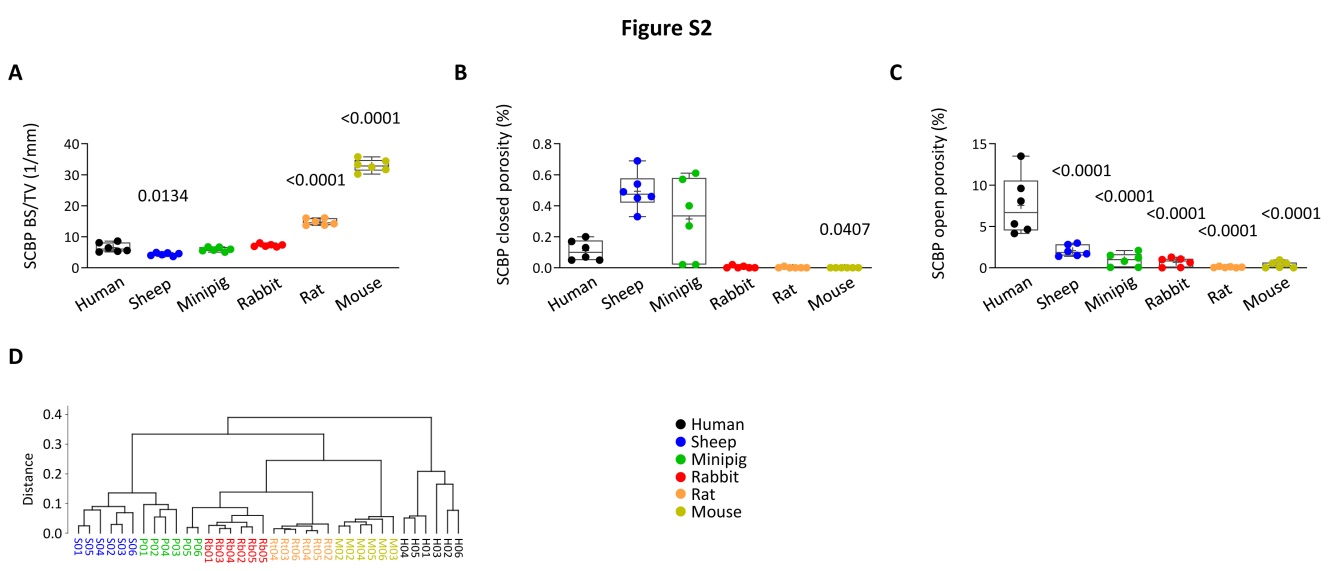
**

**Figure S2.** Comparison of the subchondral bone plate (SCBP) microstructure of human, sheep, minipig, rabbit, rat, and mouse tibial plateaus. Box plots of the SCBP **(A)** bone surface density (BS/TV), **(B)** closed, and **(C)** open porosity. **(D)** Cluster analysis of the SCBP parameters. Data points represent individual samples. *n* = 6 per species. *P* values above the box plots show comparisons to human and were determined with ANOVA or Kruskal-Wallis ANOVA.

**
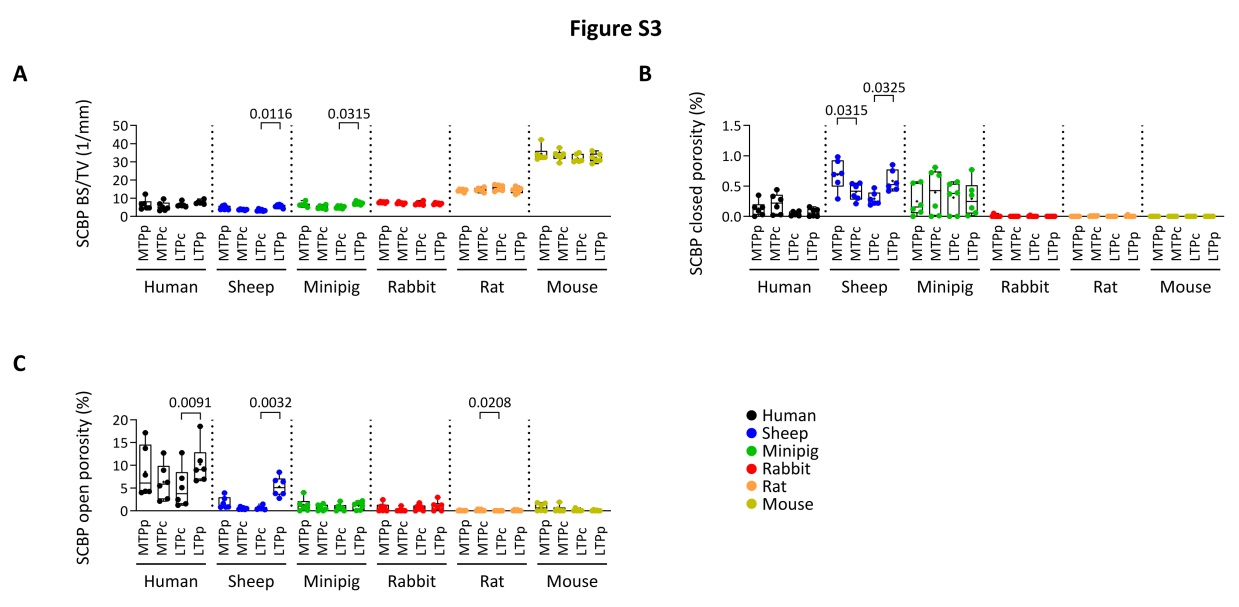
**

**Figure S3.** Detailed regional analysis of the subchondral bone plate (SCBP) microstructure in human, sheep, minipig, rabbit, rat, and mouse. Box plots of the **(A)** bone surface density (BS/TV), **(B)** closed, and **(C)** open porosity of the SCBP in four regions. Abbreviations: LTPc, lateral tibial plateau central; LTPp, lateral tibial plateau peripheral; MTPc, medial tibial plateau central; MTPp, medial tibial plateau peripheral. *n* = 6 per species. *P* values above the box plots were determined with RM-ANOVA or Friedman test. Only the relevant comparisons (i.e. MTPp vs. MTPc, LTPp vs. LTPc, MTPp vs. LTPp, MTPc vs. LTPc) were performed.

**
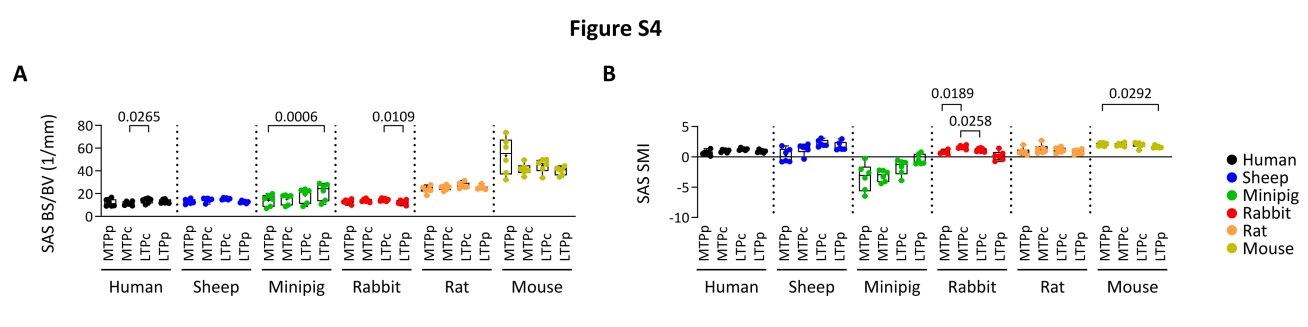
**

**Figure S4.** Detailed regional analysis of the subarticular spongiosa microstructure in human, sheep, minipig, rabbit, rat, and mouse. Box plots of the **(A)** bone surface-to-volume ratio (BS/BV) and **(B)** structure model index (SMI) of the subarticular spongiosa (SAS) in four regions. Abbreviations: LTPc, lateral tibial plateau central; LTPp, lateral tibial plateau peripheral; MTPc, medial tibial plateau central; MTPp, medial tibial plateau peripheral. *n* = 6 per species. *P* values above the box plots were determined with RM-ANOVA or Friedman test. Only the relevant comparisons (i.e. MTPp vs. MTPc, LTPp vs. LTPc, MTPp vs. LTPp, MTPc vs. LTPc) were performed.

**
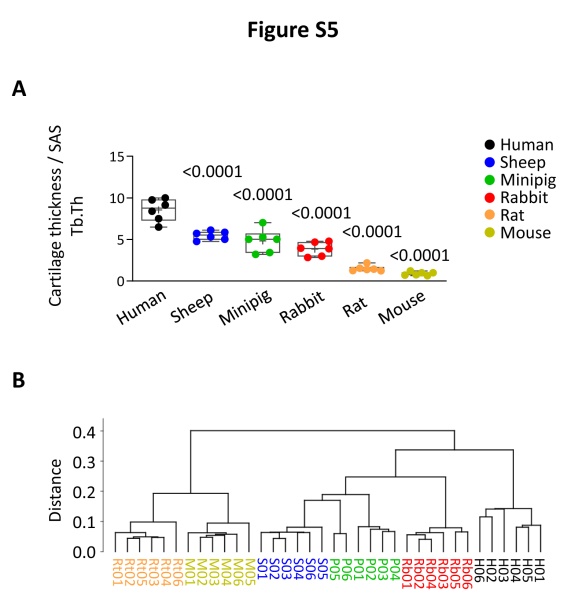
**

**Figure S5.** Evaluation of the osteochondral unit in human, sheep, minipig, rabbit, rat, and mouse. **(A)** Articular cartilage thickness normalized to the trabecular thickness (Tb.Th) of the subarticular spongiosa (SAS). *P* values above the box plots show comparisons to human and were determined with ANOVA. **(B)** Cluster analysis of all data, including cartilage thickness, tibial plateau dimensions and subchondral bone parameters, evaluated in the study. Data points represent individual samples. *n* = 6 per species.

**
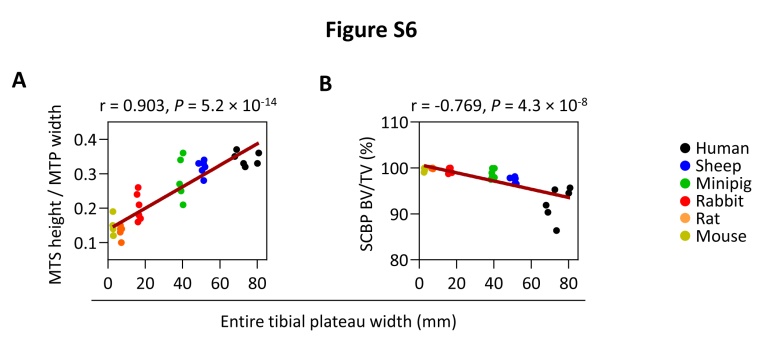
**

**Figure S6.** Correlation of tibial subchondral bone parameters with joint size. Scatter plot and linear regression of the Pearson correlation between entire tibial plateau width and **(A)** medial tibial spine (MTS) height / medial tibial plateau (MTP) width ratio, and **(B)** subchondral bone plate (SCBP) percent bone volume (BV/TV). *n* = 6 per species. *P* values and the correlation coefficients (r) are shown above the graphs.

**SUPPLEMENTARY DATA**

**Supplementary Data 1.** Raw data of the figures. Excel file containing the raw data of the box plots, multivariate analyses, and correlation analyses shown in the figures of the manuscript. For detailed description please refer to the figure legends.

**SUPPLEMENTARY BACKGROUND**

The structural organization of a knee, the most complex joint of the human body, dictates its function. The osteochondral unit, a composite of the subchondral bone and the articular cartilage^11^, guarantees its complex biomechanical movements based on an intricate morphology. The subchondral bone is composed of the subchondral bone plate, corresponding to the cortical bone of other skeletal sites, and the trabecular network of the subarticular spongiosa^11^. It is in the focus of many orthopedic and systemic conditions, including osteoarthritis (OA), osteoporosis, osteopenia, osteonecrosis, osteochondritis dissecans, and fractures^12-17^. In the knee, its local interaction is further influenced by the presence (or absence) of the fibrocartilaginous menisci, reflected in bone mineral density^18^ and microarchitectural differences among which trabecular number and thickness, anisotropy and bone sclerosis^6^.

A finding that has sparked intense interest recently is the discovery of topographical differences within individual subregions of the tibio-femoral osteochondral unit of the knee^2,3^. The closely related and clinically relevant^19,20^ concept of a location-dependent OA development^1^ underscores the urgent need to precisely reproduce pathological alterations at high quantitative analytic detail in appropriate in vivo models^4,5^, as cell cultures or ectopic in vivo models do not provide for a comparable functional joint environment capable of modeling the complex structural interplay in a standardized way^21^. While many different model animal species exist^4,5^, none show a perfect morphological agreement with the osteochondral unit of the human knee joint^4^. Moreover, the most conspicuous feature of humans which distinguishes them from all other mammalian species, the capacity of walking upright in bipedal locomotion, is not reflected at all in any of the animal models. Small animal models are ideal for screening studies and for exploring signaling pathways and pathophysiological mechanisms of the diseases^5^. The more similar size, weight and joint anatomy of larger animals to humans, make them more suited for pre-clinical studies of new therapies^4^. Comparative studies of the articular cartilage in different species^22,23^ already provide insight in the relation between its thickness and body weight^8-10^. Human subchondral bone properties have been compared to rabbits, sheep, horses^24^ and rodents^25^; however, extended cross-species data on the 3-dimensional (3D) subchondral bone architecture relevant for topographic modeling approaches are not available.

The present work aims to expand our basic understanding of the hierarchical and species-specific structure of the subchondral bone relevant for clinical and translational medicine. We performed a detailed and comprehensive analysis of its macroscopic and microstructural characteristics (Figure 1A), respecting its zonal organization. We selected the tibial plateau as model region, a highly prevalent location of OA development^26^ whose morphology is also affected by the menisci^6^. Our goal was to dissect the zonal macroscopic and microstructural characteristics of the subchondral bone in six mammalian species, considering the subchondral bone plate and subarticular spongiosa, to integrate them with cartilage thickness, compare them between species and correlate the data with joint size. We hypothesized that large animals have the most similar joint size, cartilage thickness and subchondral bone microstructure compared to humans.

**SUPPLEMENTARY METHODS**

**Study design**

The subchondral bone structure of the tibial plateau of skeletally mature young adult mice, rats, rabbits, minipigs, and sheep, the most common animal models in orthopedic research, was qualitatively and quantitatively compared with humans in a standardized fashion. The subchondral bone was first segmented into (i) the subchondral bone plate (corresponding to cortical bone) and (ii) the subarticular spongiosa (corresponding to cancellous bone) and their microstructure was evaluated at high topographic detail. (iii) Articular cartilage thickness and qualitative morphology was evaluated in four subregions. Next, these data were compared between species using principal components analysis, cluster analysis and PERMANOVA. Correlation analyses were then performed to elucidate possible links of the microstructure with the (pre-)clinically relevant joint size. A focus was on the clinically relevant standard parameters of (i) subchondral bone plate: porosity, and thickness; (ii) subarticular spongiosa: microstructural parameters such as BV/TV, BS/BV, BS/TV, Tb.Th, Tb.Sp, Tb.Pf, SMI; (iii) articular cartilage: thickness (Figure 1A).

**Samples**

On the basis of previous data^7^, a sample size of *n* = 6 was chosen. Normal human proximal tibiae (*n* = 6; including 3 females and 3 males; 4 left and 2 right knees, mean age: 65 ± 17 years) were obtained as surgical discards from the Department of Orthopaedic Surgery, Saarland University Medical Center, Homburg. Informed consent was obtained from the patients. The study was approved by the Ethics Committee of the Saarland Physicians Council (*Ärztekammer des Saarlandes, Ethik-Kommission*, No. 267/17). OA was ruled out by macroscopic and microscopic analyses. Female, skeletally mature merino sheep (mean age: 18 months, weight ~70 kg), Göttingen minipigs (age: 18-22 months, weight ~43 kg), New Zealand white rabbits (age: 8-16 months, weight ~4 kg), Wistar rats (mean age: 3.5 months, weight ~0.3 kg), and BALB/c mice (mean age: 3-4 months, weight ~0.03 kg) were sacrificed for unrelated studies, and their tibial plateaus (*n* = 6 each) were collected and frozen at -20 °C until usage. All animal experiments were conducted in accordance with the German legislation on protection of animals and were all approved by the Saarland University Animal Committee according to German guidelines (No. 43/2015).

**Regions of interests**

Length and width of the tibial plateaus were measured on sets of 2-dimensional (2D) micro-CT images. Total, medial and lateral tibial plateau width was measured on the most posterior coronal section where the tibial spines fused or reached their highest elevation^27^. Length of the tibial plateau was measured between the most posterior images where the posterior end of the medial tibial plateau was visible, and anteriorly where the lateral tibial plateau disappeared in the groove of the extensor digitorum longus muscle or, in case of human, rat and mouse, where the cartilage coverage of the plateaus ended. Height of the lateral and medial tibial spine was measured where they reached their maximal elevation on coronal 2D micro-CT sections, perpendicular to the tibial plateau width^28^.

To allow for a detailed analysis, the tibial plateaus were divided into medial peripheral, medial central, lateral central, and lateral peripheral subregions at 50% tibial plateau width. All regions of interests (ROIs) began on the most posterior coronal section where the tibial spines fused or reached their highest elevation^27^, and ended anterior from this position in a distance of 10% total tibial plateau length in human, sheep, minipig and rabbit, or at the anterior end of the tibial spines in rat and mouse.

**Micro-CT imaging**

A SkyScan 1176 micro-CT scanner (Bruker micro-CT, Kontich, Belgium) was used to scan human (90 kV tube voltage; 278 µA current; 35 µm isotropic resolution; combined 0.5 mm aluminum/copper filter; 0.4° intervals; 270 ms exposure time, 3 averaging frames), ovine, minipig (90 kV tube voltage; 278 µA current; 18 µm isotropic resolution; combined 0.5 mm aluminum/copper filter; 0.4° intervals; 270 ms exposure time, 3 averaging frames), rabbit (65 kV tube voltage; 380 µA current; 18 µm isotropic resolution; 1 mm aluminum filter; 0.4° intervals; 245 ms exposure time, 3 averaging frames), rat, and mouse (50 kV tube voltage; 500 µA current; 9 µm isotropic resolution; 0.5 mm aluminum filter; 0.3° intervals; 900ms exposure time, 3 averaging frames) tibial plateaus. After reconstruction of the datasets with NRecon (v. 1.7.0.4, Bruker micro-CT) and their identical rotation, coronal images were saved (DataViewer software v. 1.5.2.4, Bruker micro-CT)^3,27^. Volumes of interest (VOIs) of the four subregions were marked separately in the subchondral bone plate and in the subarticular spongiosa. The following parameters were determined in all VOIs using the software CTAnalyzer (v. 1.16.4.1, Bruker micro-CT): Percent bone volume (BV/TV), bone surface–to–volume ratio (BS/BV) and bone surface density (BS/TV). Percentage of closed (Po(cl)), open (Po(op)), and total pores (Po(tot)) were calculated only for the subchondral bone plate. The trabecular thickness (Tb.Th), trabecular separation (Tb.Sp), trabecular number (Tb.N), trabecular pattern factor (Tb.Pf), structure model index (SMI), degree of anisotropy (DA), fractal dimension (FD) and connectivity density (Conn.Dn) were assessed only for the subarticular spongiosa^3^. Subchondral bone plate and cartilage thickness were measured manually in three thirds (outer, middle, inner) of each subregion. For 3-dimensional (3D) reconstruction of the micro-CT image sets and modeling of trabecular thickness CTVox v. 3.2.0 (Bruker micro-CT) was used^27^. Reproducibility of micro-CT measurements was confirmed previously^3^. Data were always collected blindly regarding the subregions (and species if possible) by 3 observers (JCM, TO, SS), and decoded only after analysis.

**Histology of the osteochondral unit**

Paraffin-embedded samples were sectioned (5 µm) at constant positions within the medial and lateral tibial plateaus, and stained with Safranin O / fast green, Masson-Goldner’s trichrome and haematoxylin-eosin^29^. Pictures were obtained with an Olympus BX45 (Olympus, Hamburg, Germany) microscope and processed with Photoshop CS2 (Adobe, San José, CA, USA).

**Statistical analyses**

No outliers or other observations were removed on account of their deviation from the mean in any of the experimentation shown. To test normal distribution the Shapiro-Wilk normality test was used. When the different animal species were compared to human, depending on a normal distribution, One Way Analysis of Variance (ANOVA) followed by Dunnett’s test, or Kruskal-Wallis One Way ANOVA followed by Dunn’s test were used for determining statistical significance, and multiplicity adjusted *P* values were reported. When the relevant combinations of the four subregions were compared to each other (i.e. medial peripheral vs. medial central, lateral peripheral vs. lateral central, medial peripheral vs. lateral peripheral, medial central vs. lateral central), depending on a normal distribution, One Way Repeated Measures ANOVA (RM-ANOVA) followed by Sidak test, or the non-parametric Friedman test followed by Dunn’s test were used for determining statistical significance, and multiplicity adjusted *P* values were reported. To test correlations between osteochondral parameters and joint size, Pearson correlation coefficient (r) was calculated. All correlation coefficients were interpreted according to Evans^30^ (0.00 - 0.19 very weak; 0.20 - 0.39 weak; 0.40 - 0.59 moderate; 0.60 - 0.79 strong; 0.80 - 1.00 very strong). Multivariate analyses were performed as described previously^2,3^. Principal components analysis (PCA) with a correlation matrix routine, hierarchical cluster analysis with unweighted pair-group average (UPGMA) algorithm and Gower similarity index, and one-way permutational multivariate analysis of variance (PERMANOVA) with Gower similarity index were used, and considering multiplicity issues, the Bonferroni-corrected *P* values were reported^31^. All calculations were performed with Prism v. 8.2.1 (GraphPad Software, Inc., San Diego, CA, USA), or Past v. 4.04;^31^ *P* < 0.05 was considered statistically significant. Data was expressed as mean ± SD. Box plot diagrams always show the 75^th^ and 25^th^ percentiles (upper and lower borders of the boxes), the minimum and maximum (whiskers), the mean value (+), the median (middle line), and the individual data points (dots). Raw data of the figures are included in Supplementary Data 1.

**SUPPLEMENTARY DISCUSSION**

The present high-resolution study makes major contributions to better capture the topographical cross-species complexity of subregional differences of the subchondral bone within the osteochondral unit.

Except in humans, the subchondral bone plate thickness increased with the joint size of all animal species. Humans have an extremely thin subchondral bone plate, comparable in its thinness to that of the 20-fold lighter^5^ rabbits. This thin subchondral bone plate is specific only for the modern humans compared to non-human primates and Neanderthal fossils^32^. Curiously, it appears not to be a direct consequence of human bipedal locomotion, since a Japanese macaque trained to walk bipedally for 2-3 km daily for 8 years had a thickened subchondral bone plate^32^. In the larger species, we found higher absolute subchondral bone plate thickness, total and open porosity, and lower relative subchondral bone plate thickness, relative bone surface (BS/TV, BS/BV) indicating a less solid and compact subchondral bone plate, possibly contributing to a more efficient force distribution and nutrition towards the articular cartilage^9^. This porous structure results from different sized cavities penetrating the subchondral bone plate, including extensions of the marrow space, cylindrical canals, or small vascularized channels^33^.Thus, although the larger species resemble the human subchondral bone plate structure more faithfully than small rodents, even the more similar minipigs and rabbits are not perfect surrogates to model the extraordinarily thin and porous human subchondral bone plate.

In the subchondral bone plate, peripheral-central differences were detected in the medial tibial plateau of sheep (closed porosity) and minipig (SCBP thickness), and in the lateral tibial plateau of human (BV/TV, total porosity, open porosity), sheep (BV/TV, BS/BV, total porosity, BS/TV, closed porosity, open porosity), and minipig (SCBP thickness, BS/BV, BS/TV), suggesting a denser and more solid bone structure in the more exposed central locations that are not covered by menisci. Here, in minipigs, and in humans, the subchondral bone plate was thicker^6^. Interestingly, advanced OA disturbs this normal osteochondral pattern of submeniscal and not meniscus-covered subregions, even in a lesser loaded compartment^2^. In contrast, in small animals such an adaptation is absent. This important finding needs to be considered when aiming to dissect the role of the menisci in OA induction and progression in translational studies.

The tibial subarticular spongiosa, a region commonly affected by structural alterations in knee OA^2,3,34-37^, has a relatively denser and more complex structure in all animal species compared to humans. A possible explanation for this structural difference can be the sedentary lifestyle of modern humans, which is a significant predictor of osteopenia and osteoporosis^38^, and may be detrimental to the lower limb bone mass and structure^39,40^ in an evolutionary scale too. The more complex trabecular structure of the larger species is in good agreement with findings in a diverse range of animal species at various anatomical locations^9,24,25,41-43^. BV/TV was largely similar in animals regardless their size^42^, always higher than in humans^24^, in contrast to non-human primates, where BV/TV increased with body size^41^. In accordance, larger absolute Tb.Th^9,25,42^, and lower BS/BV^42^, Tb.N^25,43^, and Conn.Dn^43^ was reported in humans^25^ and larger animals^9,42,43^, especially when compared to rodents^25,42^. Additionally to the size and body weight of the different species, their gait, quadruped locomotion, more flexed natural hind limb position (especially in the smaller species)^4,5^, and the resulting differences in load distribution may also highly determine the microstructural adaptations and topographical patterns of the osteochondral unit. A more dense bone structure is achieved in rodents by more trabeculae, while in humans the thickness of the trabeculae is increased^25^. This is in contrast to non-human primates, where large primates have relatively thinner and more tightly packed trabeculae than small primates^41^. An important qualitative difference exists in the connectivity of trabecular elements between small vs. large animals, as trabeculae connect primarily to cortical bone in very small animals and primarily to other trabeculae in larger species^44^. Interestingly, in all species, excluding mice, FD did not differ from humans, suggesting largely similar space filling of the trabeculae. FD indicates trabecular surface complexity and has been related to tibial subchondral bone texture changes after arthroscopic partial meniscectomy in patients^45^.

Humans display the largest absolute cartilage thickness, decreasing towards species with smaller body size. Cartilage thickness increases with the body mass either in a simple^8^ or a negative allometric relationship^9,10^ (i.e. with a lower growth rate than the body as a whole) depending on the examined species, in other locations including the medial femoral condyle too^24^, while it does not correlate with collagen fiber orientation^23^.

Strong and significant correlations of osteochondral parameters with joint size confirmed previous studies^9,10,25,41,46^. Joint size, reflected here in tibial plateau width and length, was used as a surrogate for body mass, supported by a study of Malda and co-workers covering an extensive range of 58 mammalian species, where articular width in the knee joint scaled isometrically with body mass^10^. In contrast, articular cartilage and overall bone size increase with body mass in a negative allometric relationship, suggesting that these variables do not fully compensate for increasing body mass^9,10^, possibly requesting additional structural mechanisms to absorb loads in larger animals.

Consistently with our findings, in multiple other studies based on different ranges of species too, Tb.Th and Tb.Sp correlated significantly positive^25,41,46^, and BS/BV, Tb.N and Conn.Dn significantly negative with body mass^25,41,46^, while the subchondral bone plate thickness and BV/TV, and subarticular spongiosa BV/TV and DA did not correlate with body mass^10,25,46^. Inconsistently, significant positive correlations of BV/TV and negative correlations of DA with body size were reported in primates^41^. In further details, Tb.N, Tb.Th and Tb.Sp scaled with negative allometry relative to body mass^10,25^. Although, based on the evaluation of 37 species, trabecular bone features and cartilage cellularity of smaller species such as mice and rats were reported not to follow the general patterns^9^, here they also fitted well in the trends defined by joint size.

Limitations of the present study are that only morphological parameters of the osteochondral unit of one strain of each species were included, while functional, gait, cellular, and physiological characteristics were not examined^47,48^. Horses, not accepted in many countries, were not evaluated here, because the majority of equine studies examines not the stifle but the middle carpal joint or the metacarpophalangeal joint. Since entire tibial plateaus were scanned, standardization of the spatial resolution was not possible due to the extreme differences in the joint size of the species (i.e. tibial plateau width ranging between 3-74 mm). Strengths of the study include the comparison of humans to a wide range of model animal species from mice to sheep, a regional analysis with 4 subregions, with robust evaluation methods and multivariate statistics.

This study has important translational implications. The order of similarity of both macroscopic and microanatomy of the subchondral bone of the tibial plateau to humans was: sheep ≈ minipigs > rabbits >> rats >> mice, supporting the applicability of these species as animal models for studies of subchondral bone alterations. Although availability and surgical requirements may favor small animals^4,5^, anatomical and pathophysiological similarities shift the balance towards larger animals in true preclinical studies. When resemblance of the extremely thin and porous subchondral bone plate of humans is a key requirement, minipigs and rabbits are the best choice. If the characteristic topographical patterns according to the meniscal coverage are of importance like in the clinically relevant models of OA based on (partial) meniscectomy, only sheep and minipigs reflect the human situation. The structurally weaker cancellous subchondral bone of humans is matched by none of the examined species, although minipigs, rabbits, and sheep offer acceptable compromises. Should the planned experiment require a faithful mirroring of lateral-to-medial differences, sheep or rats may be chosen. Sheep appear to be better suited for precision surgical interventions altering medio-lateral load distribution^7^. The marked differences of small rodents discourage their use as realistic models of human subchondral trabecular structure and structural patterns related to meniscal coverage. Our spatial analyses may serve as a basis for a variety of forthcoming experimental designs, for example integrating genetic, environmental or functional factors into these insights of osteochondral tissue organization.

In sum, this comparative investigation addresses major gaps in our understanding of topographical patterning of the subchondral bone that are critical for clinical and translational medical approaches and may serve to provide new directions for future research.

**SUPPLEMENTARY REFERENCES**

11. Goldring SR, Goldring MB. Changes in the osteochondral unit during osteoarthritis: structure, function and cartilage-bone crosstalk. *Nat Rev Rheumatol*. Nov 2016;12(11):632-644. doi:10.1038/nrrheum.2016.148

12. Madry H, van Dijk CN, Mueller-Gerbl M. The basic science of the subchondral bone. *Knee Surg Sports Traumatol Arthrosc*. Apr 2010;18(4):419-33. doi:10.1007/s00167-010-1054-z

13. Stewart HL, Kawcak CE. The Importance of Subchondral Bone in the Pathophysiology of Osteoarthritis. *Front Vet Sci*. 2018;5:178. doi:10.3389/fvets.2018.00178

14. Boyde A. The Bone Cartilage Interface and Osteoarthritis. *Calcif Tissue Int*. Sep 2021;109(3):303-328. doi:10.1007/s00223-021-00866-9

15. Katz JN, Arant KR, Loeser RF. Diagnosis and Treatment of Hip and Knee Osteoarthritis: A Review. *JAMA*. Feb 9 2021;325(6):568-578. doi:10.1001/jama.2020.22171

16. Lepage SIM, Robson N, Gilmore H, et al. Beyond Cartilage Repair: The Role of the Osteochondral Unit in Joint Health and Disease. *Tissue Eng Part B Rev*. Apr 2019;25(2):114-125. doi:10.1089/ten.TEB.2018.0122

17. Jacob G, Shimomura K, Nakamura N. Osteochondral Injury, Management and Tissue Engineering Approaches. *Front Cell Dev Biol*. 2020;8:580868. doi:10.3389/fcell.2020.580868

18. Sannmann F, Laredo JD, Chappard C, Engelke K. Impact of meniscal coverage on subchondral bone mineral density of the proximal tibia in female subjects - A cross-sectional in vivo study using QCT. *Bone*. May 2020;134:115292. doi:10.1016/j.bone.2020.115292

19. Hochberg MC, Guermazi A, Guehring H, et al. Effect of Intra-Articular Sprifermin vs Placebo on Femorotibial Joint Cartilage Thickness in Patients With Osteoarthritis: The FORWARD Randomized Clinical Trial. *JAMA*. Oct 8 2019;322(14):1360-1370. doi:10.1001/jama.2019.14735

20. Lohmander LS, Hellot S, Dreher D, et al. Intraarticular sprifermin (recombinant human fibroblast growth factor 18) in knee osteoarthritis: a randomized, double-blind, placebo-controlled trial. *Arthritis Rheumatol*. Jul 2014;66(7):1820-31. doi:10.1002/art.38614

21. Cai X, Daniels O, Cucchiarini M, Madry H. Ectopic models recapitulating morphological and functional features of articular cartilage. *Annals of Anatomy - Anatomischer Anzeiger*. 2021/09/01/ 2021;237:151721. doi:https://doi.org/10.1016/j.aanat.2021.151721

22. Frisbie DD, Cross MW, McIlwraith CW. A comparative study of articular cartilage thickness in the stifle of animal species used in human pre-clinical studies compared to articular cartilage thickness in the human knee. *Vet Comp Orthop Traumatol*. 2006;19(3):142-6.

23. Kaab MJ, Gwynn IA, Notzli HP. Collagen fibre arrangement in the tibial plateau articular cartilage of man and other mammalian species. *J Anat*. Jul 1998;193 ( Pt 1):23-34. doi:10.1017/s0021878298003744

24. Chevrier A, Kouao AS, Picard G, Hurtig MB, Buschmann MD. Interspecies comparison of subchondral bone properties important for cartilage repair. *J Orthop Res*. Jan 2015;33(1):63-70. doi:10.1002/jor.22740

25. Barak MM, Lieberman DE, Hublin JJ. Of mice, rats and men: trabecular bone architecture in mammals scales to body mass with negative allometry. *J Struct Biol*. Aug 2013;183(2):123-31. doi:10.1016/j.jsb.2013.04.009

26. Ledingham J, Regan M, Jones A, Doherty M. Radiographic patterns and associations of osteoarthritis of the knee in patients referred to hospital. *Ann Rheum Dis*. Jul 1993;52(7):520-6. doi:10.1136/ard.52.7.520

27. Olah T, Reinhard J, Gao L, Goebel LKH, Madry H. Reliable landmarks for precise topographical analyses of pathological structural changes of the ovine tibial plateau in 2-D and 3-D subspaces. *Sci Rep*. Jan 8 2018;8(1):75. doi:10.1038/s41598-017-18426-3

28. Tan AL, Baboolal T, Benjamin M, Yonenaga T, Binks D, McGonagle D. SAT0435 Tibial Spine Morphology and Association with Anterior Cruciate Ligament Degeneration – A Combined MRI, X-rays and Histology Study of Early Knee Osteoarthritis. *Annals of the Rheumatic Diseases*. 2016;75(Suppl 2):828-829. doi:10.1136/annrheumdis-2016-eular.5728

29. Orth P, Peifer C, Goebel L, Cucchiarini M, Madry H. Comprehensive analysis of translational osteochondral repair: Focus on the histological assessment. *Prog Histochem Cytochem*. Oct 2015;50(3):19-36. doi:10.1016/j.proghi.2015.10.001

30. Evans JD. *Straightforward statistics for the behavioral sciences*. Straightforward statistics for the behavioral sciences. Thomson Brooks/Cole Publishing Co; 1996:xxii, 600-xxii, 600.

31. Hammer Ø, Harper DAT, Ryan PD. PAST: Paleontological statistics software package for education and data analysis. *Palaeontologia Electronica*. 2001;4(1):1-9.

32. Mazurier A, Nakatsukasa M, Macchiarelli R. The inner structural variation of the primate tibial plateau characterized by high-resolution microtomography. Implications for the reconstruction of fossil locomotor behaviours. *Comptes Rendus Palevol*. 2010/09/01/ 2010;9(6):349-359. doi:https://doi.org/10.1016/j.crpv.2010.07.020

33. Ching K, Houard X, Berenbaum F, Wen C. Hypertension meets osteoarthritis - revisiting the vascular aetiology hypothesis. *Nat Rev Rheumatol*. Sep 2021;17(9):533-549. doi:10.1038/s41584-021-00650-x

34. Rapagna S, Roberts BC, Solomon LB, Reynolds KJ, Thewlis D, Perilli E. Tibial cartilage, subchondral bone plate and trabecular bone microarchitecture in varus- and valgus-osteoarthritis versus controls. *J Orthop Res*. Sep 2021;39(9):1988-1999. doi:10.1002/jor.24914

35. Han X, Cui J, Xie K, et al. Association between knee alignment, osteoarthritis disease severity, and subchondral trabecular bone microarchitecture in patients with knee osteoarthritis: a cross-sectional study. *Arthritis Res Ther*. Sep 4 2020;22(1):203. doi:10.1186/s13075-020-02274-0

36. Shiraishi K, Chiba K, Okazaki N, et al. In vivo analysis of subchondral trabecular bone in patients with osteoarthritis of the knee using second-generation high-resolution peripheral quantitative computed tomography (HR-pQCT). *Bone*. Mar 2020;132:115155. doi:10.1016/j.bone.2019.115155

37. Janvier T, Jennane R, Toumi H, Lespessailles E. Subchondral tibial bone texture predicts the incidence of radiographic knee osteoarthritis: data from the Osteoarthritis Initiative. *Osteoarthritis Cartilage*. Dec 2017;25(12):2047-2054. doi:10.1016/j.joca.2017.09.004

38. Braun SI, Kim Y, Jetton AE, Kang M, Morgan DW. Sedentary Behavior, Physical Activity, and Bone Health in Postmenopausal Women. *J Aging Phys Act*. Apr 2017;25(2):173-181. doi:10.1123/japa.2016-0046

39. Weaver CM, Gordon CM, Janz KF, et al. The National Osteoporosis Foundation's position statement on peak bone mass development and lifestyle factors: a systematic review and implementation recommendations. *Osteoporos Int*. Apr 2016;27(4):1281-1386. doi:10.1007/s00198-015-3440-3

40. Koedijk JB, van Rijswijk J, Oranje WA, et al. Sedentary behaviour and bone health in children, adolescents and young adults: a systematic review-supplementary presentation. *Osteoporos Int*. Oct 2017;28(10):3075-3076. doi:10.1007/s00198-017-4195-9

41. Ryan TM, Shaw CN. Trabecular bone microstructure scales allometrically in the primate humerus and femur. *Proc Biol Sci*. May 7 2013;280(1758):20130172. doi:10.1098/rspb.2013.0172

42. Mullender MG, Huiskes R, Versleyen H, Buma P. Osteocyte density and histomorphometric parameters in cancellous bone of the proximal femur in five mammalian species. *J Orthop Res*. Nov 1996;14(6):972-9. doi:10.1002/jor.1100140618

43. Bagi CM, Berryman E, Moalli MR. Comparative bone anatomy of commonly used laboratory animals: implications for drug discovery. *Comp Med*. Feb 2011;61(1):76-85.

44. Swartz SM, Parker A, Huo C. Theoretical and empirical scaling patterns and topological homology in bone trabeculae. *J Exp Biol*. Feb 1998;201(Pt 4):573-90.

45. Wolski M, Thorlund JB, Stachowiak GW, et al. Early tibial subchondral bone texture changes after arthroscopic partial meniscectomy in knees without radiographic OA: A prospective cohort study. *J Orthop Res*. Aug 2020;38(8):1819-1825. doi:10.1002/jor.24593

46. Doube M, Klosowski MM, Wiktorowicz-Conroy AM, Hutchinson JR, Shefelbine SJ. Trabecular bone scales allometrically in mammals and birds. *Proc Biol Sci*. Oct 22 2011;278(1721):3067-73. doi:10.1098/rspb.2011.0069

47. Lajeunesse D, Reboul P. Subchondral bone in osteoarthritis: a biologic link with articular cartilage leading to abnormal remodeling. *Curr Opin Rheumatol*. Sep 2003;15(5):628-33. doi:10.1097/00002281-200309000-00018

48. Mahjoub M, Berenbaum F, Houard X. Why subchondral bone in osteoarthritis? The importance of the cartilage bone interface in osteoarthritis. *Osteoporos Int*. Dec 2012;23 Suppl 8:S841-6. doi:10.1007/s00198-012-2161-0
